# MicroRNA‐155‐5p suppresses PD‐L1 expression in lung adenocarcinoma

**DOI:** 10.1002/2211-5463.12853

**Published:** 2020-04-22

**Authors:** Jiansheng Huang, Qiaoyou Weng, Yang Shi, Weibo Mao, Zhigang Zhao, Rongzhen Wu, Jianmin Ren, Shiji Fang, Chenying Lu, Yongzhong Du, Jiansong Ji

**Affiliations:** ^1^ Key Laboratory of Imaging Diagnosis and Minimally Invasive Intervention Research Lishui Hospital of Zhejiang University China; ^2^ School of Pharmacy Zhejiang University Hangzhou China

**Keywords:** lung adenocarcinoma, MiR‐155‐5p, miRNA, PD‐L1

## Abstract

MiR‐155‐5p is a key oncogenic microRNA that maintains immune homeostasis and mediates cross‐talk between inflammation and tumorigenesis. High expression of programmed death ligand‐1 (PD‐L1) also plays an important role in immune tolerance in tumors. The present study aimed to explore the relationship between miR‐155‐5p and PD‐L1 in lung adenocarcinoma (LUAD) cells A549 and H1650. The expression levels of miR‐155‐5p and PD‐L1 in LUAD patients were detected by a quantitative reverse transcriptase‐polymerase chain reaction (qRT‐PCR) and mimics of miR‐155‐5p were used to model increased expression in A549 or H1650 cells. After 24 h, we measured levels of PD‐L1 by qRT‐PCR, western blotting and flow cytometry. In addition, we identified two sites in the PD‐L1 3′‐UTR (5′‐AGCAUUA‐3′ and 5′‐GCAUUAA‐3′) that can be bound by miR‐155‐5p using TargetScan (http://www.targetscan.org). Compared to normal tissue, miR‐155‐5p was overexpressed in tumor tissue (*P* = 0.0456), whereas the expression of PD‐L1 was not significantly different (*P* = 0.1349). The expression levels of miR‐155‐5p and PD‐L1 were negatively correlated (*r* = −0.6409, *P* = 0.0459 and *r* = −0.7544, *P* = 0.0117). Exogenous overexpression of miR‐155‐5p decreased the mRNA, total protein and membrane protein expression levels of PD‐L1 both in A549 and H1650 cells (*P* < 0.05). Taken together, our data suggest that miR‐155‐5p may suppress the expression of PD‐L1 in LUAD.

## Graphical abstract

The present study aimed to verify the regulation of programmed death ligand‐1 (PD‐L1) expression by miR155‐5p in lung adenocarcinoma. We conducted experiments at tissue and cell levels, and analyzed the potential mechanism through bioinformatics. The results show that miR‐155‐5p overexpression can inhibit PD‐L1 mRNA and protein expression. The mechanism may be that miR‐155‐5p affects PD‐L1 translation by binding to 3′‐UTR.

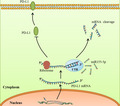

**Abbreviations**
IFN: interferon
LUAD: lung adenocarcinoma
miRNAs: microRNAs
PD‐L1: programmed cell death ligand‐1

MicroRNAs (miRNAs) are non‐protein‐encoding small RNAs of approximately 22 nucleotides in length that regulate target gene expression at the post‐transcriptional level [1, 2]. Collectively, miRNA genes are one of the most abundant classes of regulatory genes in mammals, and deregulated miRNAs play an important role in human diseases such as cancer [3, 4]. It has been shown that miR‐155‐5p is highly expressed in many cancers, such as lung cancer, breast cancer, colon cancer, lymphoma and other tumors [5]. Moreover, the high expression of miR‐155‐5p has been found to correlate with poor prognoses of multiple cancers, such as lung cancer and cervical cancer [6, 7]. Programmed death ligand‐1 (PD‐L1), also known as B7‐H1 or CD274, is constitutively expressed in T cells, B cells, dendritic cells, macrophages and mesenchymal stem cells [8]. Meanwhile, PD‐L1 was overexpressed in various human cancers, and it was found to play a central role in the immune response of tumors [9, 10].

Currently, several studies have focused on the relationship between miR‐155‐5p and PD‐L1 in cancer. In human dermal lymphatic endothelial cells, miR‐155‐5p was able to affect the kinetics of PD‐L1 and reduce its expression upon interferon (IFN)‐γ and tumor necrosis factor‐α treatment via directly binding to the 3′‐UTR of PD‐L1 [11]. However, in lymphoma cells, miR‐155‐5p could positively regulate the transcriptional activity of PD‐L1 and inhibit CD8^+^ T cell function via the PD1/PD‐L1 pathway to enhance the immune tolerance of tumor cells [12]. The contradictory findings of such studies may be the result of different experimental subjects and conditions, suggesting that the regulation of PD‐L1 by miR‐155‐5p is different in different diseases or under different conditions. Therefore, it is necessary to investigate the relationship between PD‐L1 and miR‐155‐5p under certain conditions.

Data regarding the effect of miR‐155‐5p on PD‐L1 in lung adenocarcinoma (LUAD) remain limited. In the present study, we found that overexpression of miR‐155‐5p in A549 cells resulted in the suppression of PD‐L1 expression at the mRNA, total protein and membrane protein levels. Furthermore, overexpression of miR‐155‐5p also resulted in a significant reduction of IFN‐γ‐induced PD‐L1 expression. Bioinformatics analysis shown that there are two miR‐155‐5p binding sites in the 3′‐UTR of PD‐L1. Overall, the present study reveals the relationship between miR‐155‐5p and PD‐L1 in LUAD cells and provides new insights into the association between inflammation, cancer and the immune response.

## Materials and methods

### Cell culture

A549 and H1650 cells were purchased from the Shanghai Institute for Biological Sciences, Chinese Academy of Sciences (Shanghai, China). Cells were cultured in RPMI 1640 medium (Gibco, Gaithersburg, MD, USA) supplemented with 10% fetal bovine serum (HyClone, Logan, UT, USA) and 1% antimycotic‐antibiotic solution (Beijing Solarbio, Beijing, China). Cells were kept in a constant‐temperature incubator of 5% CO_2_ and 37 °C.

### RNA oligonucleotide and cell transfection

MiR‐155 mimics and negative control (NC) oligonucleotides were purchased from Shanghai GenePharma Co., Ltd. (Shanghai, China). Cells were transfected with a specific concentration (2.5 nm, 5 nm, 10 nm and 20 nm or 5 nm, 10 nm, 20 nm and 40 nm) of miR‐155‐5p mimics, NC 20 nm) for 24 h using Lipofectamine® 3000 (Invitrogen, Carlsbad, CA, USA) in accordance with the manufacturer's instructions. In another experiment, after 24 h of transfection, cells were stimulated with IFN‐γ for 6 h and harvested for analysis.

### RNA extraction and quantitative reverse transcriptase‐polymerase chain reaction (qRT–qPCR)

Total RNA was extracted from cells and tissues using TRIzol reagent (Invitrogen) in accordance with the manufacturer's instructions. mRNA and miRNA were reverse transcribed (37 °C for 15 min, 85 °C for 5 s, final storage at 4 °C) using a PrimeScript™ RT reagent kit (Takara Bio Inc., Otsu, Japan) and qPCR was performed with SYBR® Premix Ex Taq™ (Shanghai Yeasen, Shanghai, China). The sequences of miR‐155‐5p special RT primers were 5′‐GTCGTATCCAGTGCGTGTCGTGGAGTCGGCAATTGCACTGGATACGACCCCCTA‐3′. The sequences of quantitative PCR primers were: miR‐155‐5p F‐5′‐GGGTTAATGCTAATCGTGATA‐3′ and R‐5′‐CAGTGCGTGTCGTGGAGT‐3′; U6 F‐5′‐CTCGCTTCGGCAGCACA‐3′ and R‐5′‐AACGCTTCACGAATTTGCGT‐3′ [13]; PD‐L1 F‐5′‐GTGGCATCCAAGATACAAACTCAA‐3′ and R‐5′‐TCCTTCCTCTTGTCACGCTCA‐3′; and β‐actin F‐5′‐AGCAAGCAGGAGTATGACG‐3′ and R‐5′‐GTGGGGTGGCTTTTAGGA‐3′.

### Western blot analysis

Briefly, whole cell was lysed with radio‐immunoprecipitation assay buffer (Beyotime, Shanghai, China) and separated using 10% SDS/PAGE [14]. Proteins were transferred and the membranes (Merck Millipore, Carrigtwohill, Ireland) were blocked at room temperature. Then, the anti‐PD‐L1 (dilution 1 : 2000; Abcam, Cambridge, MA, USA) or anti‐β‐actin (dilution 1 : 2000; Beyotime) antibodies were added to bind to the target proteins. Finally, horseradish peroxidase ‐conjugated secondary antibodies were added and the signals were visualized using an ECL Kit (Beyotime).

### Flow cytometry analysis

The cells were collected and stained for 30 min with a PE‐conjugated anti‐PD‐L1 antibody (20 µL per test; BD Pharmingen, San Diego, CA, USA) that was diluted in phopshate‐buffered saline. Labeled cells were analyzed via flow cytometry (FC 500 MCL; Beckman Coulter, Fullerton, CA, USA). The data were analyzed using flowjo (https://www.flowjo.com) and the levels of cell‐surface PD‐L1 protein were estimated by the average fluorescence intensity and rate of positivity. The experiments were performed at least three times.

### Immunohistochemistry and outcome determination

Briefly, the sections were deparaffinized in xylene and rehydrated in alcohol and water. The antigens were retrieved using citrate buffer in water bath, and the endogenous peroxidase activity was blocked by hydrogen peroxide. Then the slides were incubated with primary monoclonal antibody targeting the PD‐L1 at 4 °C overnight. After binding to horseradish peroxidase‐conjugated secondary antibody, the PD‐L1 protein was detected using a visual grading system and semi‐quantified based on the H‐scoring method [15]. According to the results of immunohistochemistry experiments, the immunohistochemistry scores of PD‐L1 expression of tumor cells in the specimens were set to 0, 1, 2, 3, 4 and 5. The corresponding proportion of PD‐L1‐positive tumor cells is < 1%, 1–5%, 5–10%, 10–20%, 20–50% and > 50%.

### Statistical analysis

The results are reported as the mean ± SD. The difference among the groups was estimated by Student's *t*‐tests. Statistical analyses were performed with spss, version 22.0 (IBM Corp., Armonk, NY, USA).

## Results

### The PD‐L1 3′‐UTR contains predicted target sites for miR‐155‐5p

**Fig. 1:**
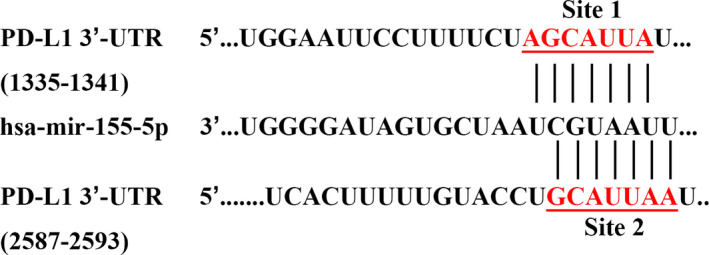
The PD‐L1 3′‐UTR contains predicted target sites for miR‐155‐5p. As predicted by TargetScan, there are two binding sites in the PD‐L1 3′‐UTR that can be recognized by miR‐155‐5p.

We analyzed the potential binding sites using the miRNA target gene prediction tool TargetScan (http://www.targetscan.org). The results revealed two potential miR‐155‐5p binding sites in the 3′‐UTR of PD‐L1 (representative transcript: ENST00000381573.4). The sequences of two sites are 5′‐AGCAUUA‐3′ and 5′‐GCAUUAA‐3′ (Fig. 1).

### Correlation between miR‐155‐5p and PD‐L1 in LUAD

**Fig. 2:**
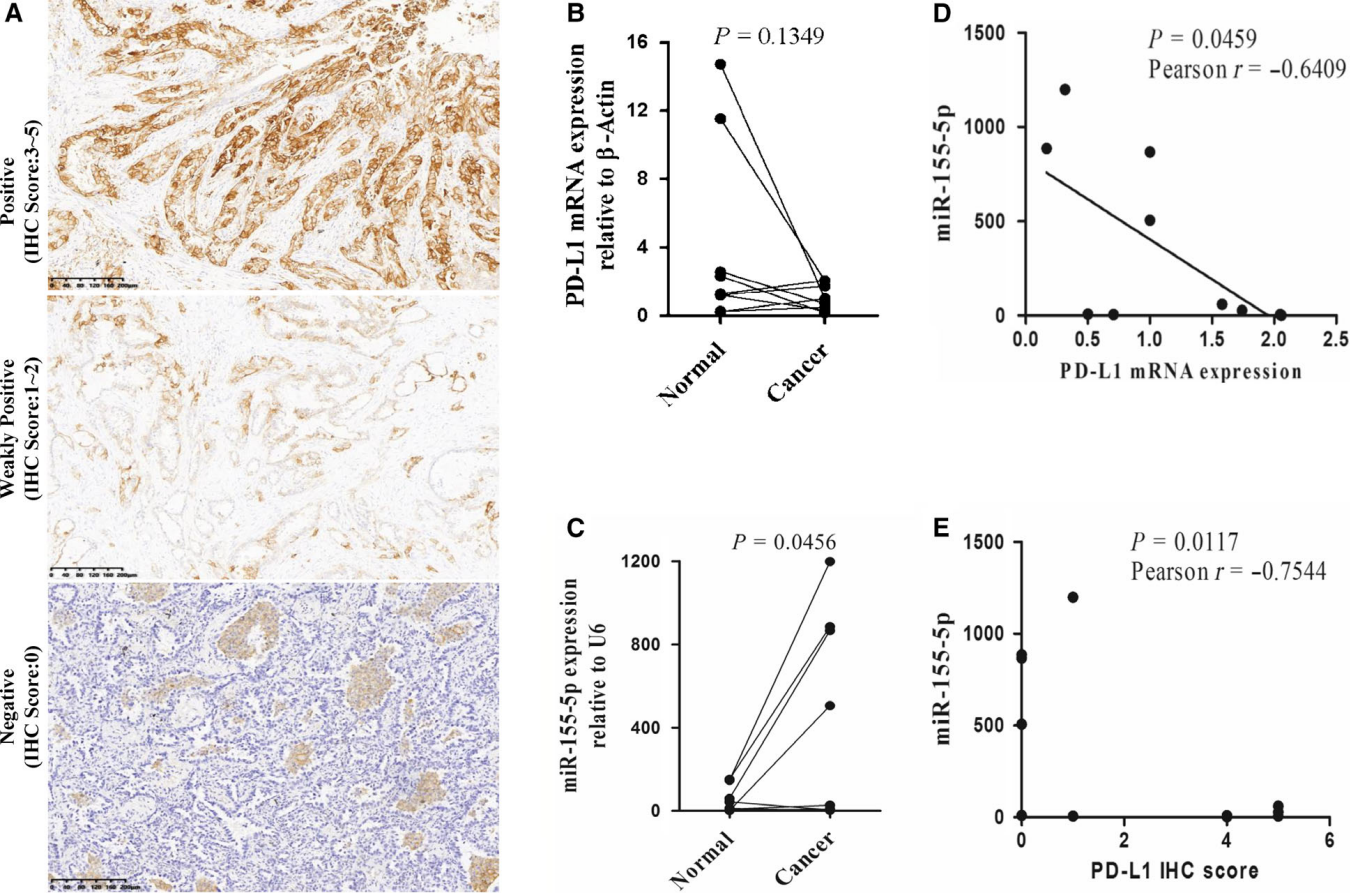
The expression levels of miR‐155‐5p and PD‐L1 were negatively correlated in LUAD. (A) Representative immunohistochemistry staining for PD‐L1 in tumor tissues of an LUAD patient. Scale bars = 200 µm. (B) The expression of PD‐L1 mRNA in tumor tissues and adjacent tissues. β‐actin acted as the endogenous control (*n* = 9, *P* = 0.1349). (C) The expression of miR‐155‐5p in tumor tissues and adjacent tissues. U6 acted as the endogenous control (*n* = 9, *P* = 0.0456). (D) Correlation between miR‐155‐5p and PD‐L1 mRNA expression in cancer tissues (*r* = −0.6409, *P* = 0.0459). (E) Correlation between miR‐155‐5p and PD‐L1 immunohistochemical score in cancer tissues (*r* = −0.7544, *P* = 0.0117). Statistical analysis was performed using an unpaired Student's *t*‐test with linear regression.

First, we evaluated the protein levels by immunohistochemistry to determine the expression of PD‐L1 in LUAD (Fig. 2A). PD‐L1 immunohistochemical results of 74 LUAD samples showed that 60 cases were negative, four cases were weakly positive and 10 cases were strongly positive, with the rate of positivity being 18.9% (14/74). Then, miR‐155‐5p and PD‐L1 mRNA expression were measured in nine LUAD samples and the corresponding adjacent tissues by qRT‐PCR. PD‐L1 mRNA expression was downregulated in five tumor tissues and upregulated in four tumor tissues compared to expression in adjacent tissues (*P* = 0.1349) (Fig. 2B). MiR‐155‐5p expression was significantly increased in tumor tissue (*P* = 0.0456) (Fig. 2C). Next, we analyzed the correlation between the expression levels of miR‐155‐5p and PD‐L1 in LUAD. The results showed that the expression of miR‐155‐5p and PD‐L1 is negatively correlated (*r* = −0.6409, *P* = 0.0459 and *r* = −0.7544, *P* = 0.0117) (Fig. 2D,E), suggesting that miR‐155‐5p may negatively regulate PD‐L1.

### miR‐155‐5p suppresses the expression of PD‐L1 in LUAD cells

**Fig. 3:**
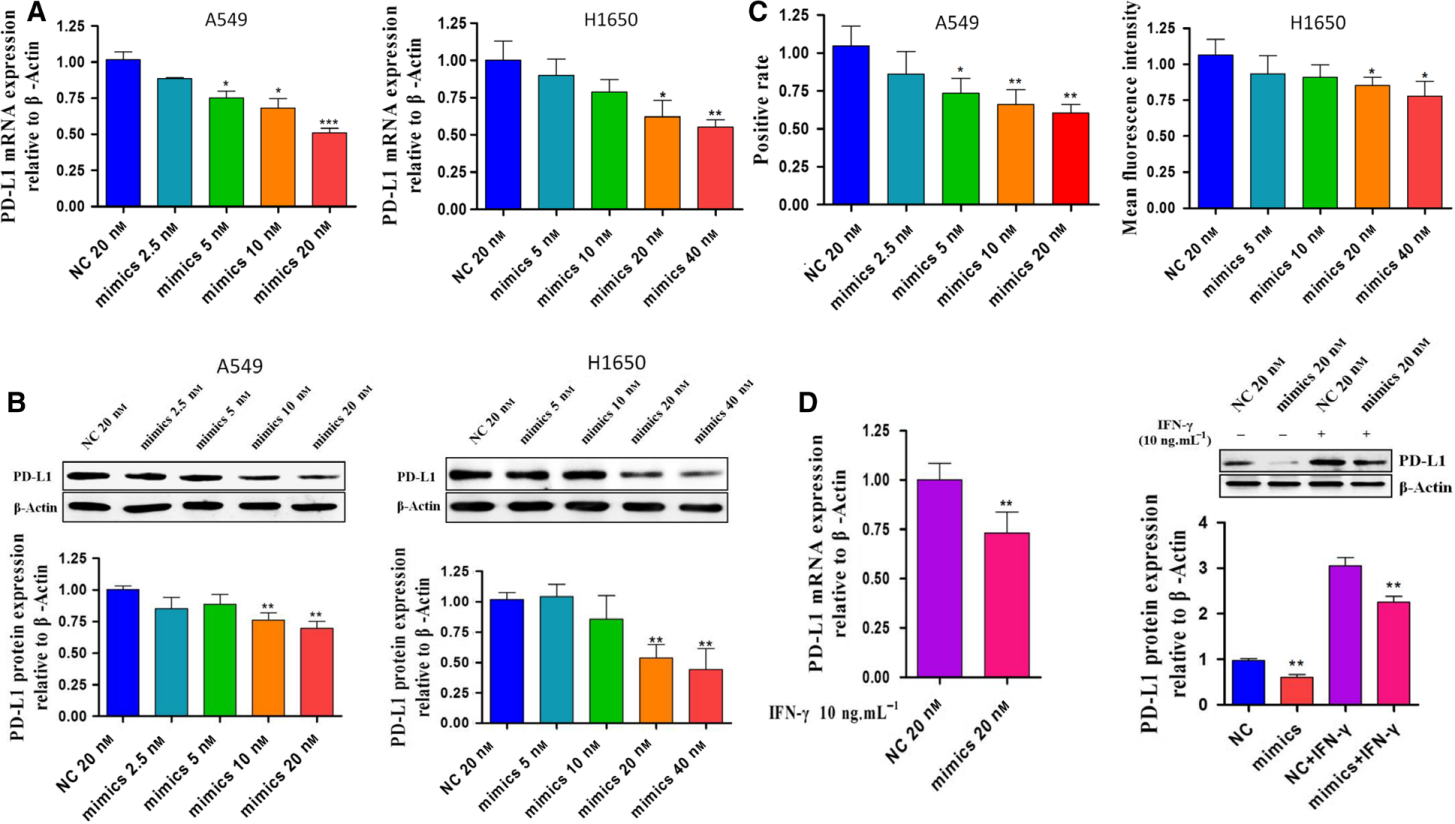
miR‐155‐5p suppresses the expression of PD‐L1. (A) PD‐L1 mRNA expression (mean ± SD) was measured by RT‐PCR after overexpression of miR‐155‐5p in A549 and H1650 cells. β‐actin was the endogenous control. (B) PD‐L1 expression (mean ± SD) was measured by western blotting after overexpression of miR‐155‐5p in A549 and H1650 cells. β‐actin was the endogenous control. (C) The expression of PD‐L1 membrane protein in A549 and H1650 cells was detected by flow cytometry and the result was shown as positive rate and median fluorescence intensity. (D) mRNA and protein expression of PD‐L1 following IFN‐γ stimulation (10 ng·mL^−1^, 6 h) in A549 cells transfected with the miR‐155‐5p mimic (20 nm, 24 h). β‐actin was the endogenous control. Statistical analysis was performed using an unpaired Student's *t*‐test (*P < 0.05, **P < 0.01, ***P < 0.001). Each experiment was independently repeated at least three times.

To examine the effect of miR‐155‐5p on endogenous PD‐L1 expression, we transfected the LUAD cell line A549 and H1650 with miR‐155‐5p mimics with increasing concentration (2.5, 5, 10 and 20 nm in A549 and 5, 10, 20 and 40 nm in H1640) and its negative control (20 nm) for 24 h. Then, we detected the expression of PD‐L1 by qRT‐PCR, western blotting and flow cytometry. The results showed that, when miR‐155‐5p was overexpressed, the mRNA, total protein and membrane protein levels of PD‐L1 decreased by approximately 30–40% (*P* < 0.05) (Fig. 3A–C), which suggested that PD‐L1 is negatively regulated by miR‐155‐5p. To further confirm this function of miR‐155‐5p, we added IFN‐γ to stimulate PD‐L1 expression after transfecting miR‐155‐5p mimics in A549. The results showed that upregulation of miR‐155‐5p resulted in significant attenuation of IFN‐γ‐induced PD‐L1 expression (*P* < 0.05) (Fig. 3D).

## Discussion

In terms of both incidence and mortality, lung cancer is the most common cancer in the world [16]. Adenocarcinoma by itself accounts for the majority of lung cancer because its incidence has gradually surpassed lung squamous cell carcinoma in recent years [17]. Therefore, it is necessary to thoroughly understand the molecular regulation mechanisms driving LUAD. In the present study, we revealed that the expression of PD‐L1 is negatively correlated with the expression of miR‐155‐5p in LUAD. Overexpression of miR‐155‐5p in the LUAD cell line A549 downregulated the expression of PD‐L1 at both mRNA and protein levels. We identified miR‐155‐5p as a post‐transcriptional PD‐L1 regulator that inhibits the levels of PD‐L1 expression in LUAD. Other miRNAs, such as miR‐34 or miR‐138‐5p [18, 19], have been shown to be involved in the direct regulation of PD‐L1 expression in different tumors, suggesting that miRNA regulation of PD‐L1 is a ubiquitous phenomenon in tumors.

PD‐L1 is one of the key molecules in mediating tumor immune evasion. PD‐L1 that is expressed in cancer cells could specifically bind to the receptor (PD‐1) on the surface of activated T cells in the tumor microenvironment and transmit negative regulatory signals to inhibit T cell activation, proliferation or cytokine secretion [20, 21]. The rates of PD‐L1 expression are quite different (25–65%) in non‐small‐cell lung cancer as determined by immunohistochemistry [22, 23, 24]. The difference may be a result of various factors, such as the regional specificity of the tumor, differences in the experimental method or criteria for judgment, or the pathological state of the patient. To date, studies on the expression of PD‐L1 in LUAD are still insufficient and experience the same problems. Yang *et al*. [25] showed that PD‐L1 is overexpressed in 39.9% (65/163) of stage I LUAD patients, and the expression level is closely related to high‐grade differentiation and vascular invasion. In the present study, we analyzed the expression of PD‐L1 by immunohistochemistry in 74 patients with LUAD. There were only 14 (18.9%) cases of PD‐L1 positive expression. This is consistent with the value of 17% reported in the study by Janzic *et al*. [26]. In addition, we noted that, in non‐squamous non‐small‐cell lung cancer, PD‐L1 positivity is associated with the response to PD‐1 inhibitor therapy [27]. However, the reported objective response rate of PD‐1 and CTLA‐4 pathway inhibitors in lung cancer is 10–20% [28]. This suggests that the low rate of PD‐L1‐positive LUAD may be one reason for the low response rate of LUAD to monoclonal antibody therapy.

MiR‐155‐5p is located within a region known as the B cell integration cluster and is highly expressed in activated B cells, T cells and other immune cells [29]. It is an important oncogenic microRNA that is a marker of inflammation and tumors and is correlated with immune homeostasis [30, 31]. We observed that overexpression of miR‐155‐5p decreased PD‐L1 expression in A549 and H1650 cells. Moreover, miR‐155‐5p also affected PD‐L1 expression after IFN‐γ stimulation. This indicates that the regulation of PD‐L1 by miR‐155‐5p is likely to occur at the post‐transcriptional level. We used predictive software analysis to show that there are two binding sites for miR‐155‐5p in the 3′‐UTR of PD‐L1, which is consistent with other the results of other studies [11]. Few studies have shown a direct relationship between miR‐155‐5p and PD‐L1 in cancer. However, in normal cells, such as dermal vascular and stromal cells, miR‐155‐5p under the stimulation of cytokines acts to suppress PD‐L1 gene expression by directly binding related sites. Therefore, we speculate that, in LUAD cells, miR‐155‐5p also regulates PD‐L1 in this manner.

Generally, the present study provides a novel understanding of the post‐transcriptional regulation of PD‐L1 in LUAD. We hypothesized that miR‐155‐5p was involved in the immune response of LUAD; the cross‐talk between miR‐155 and PD‐L1 may provide a new mechanism for inflammation‐associated tumorigenesis and suggests a potential use for miR‐155‐5p and PD‐L1 in LUAD therapy.

## Conflict of interests

The authors declare that they have no conflicts of interest.

## Author contributions

YD, JJ, ZZ and JH conceived and designed the project. JH, QW, JR, WM and YS performed the experimental work. JH, QW, YS, RW and ZZ wrote the manuscript, researched the data, reviewed and edited the manuscript. YD, JJ, ZZ, JH, QW, RW, JR, SF, CL and YS contributed to the discussion.
